# Supplementary material for: Genomic Characterization and Environmental Distribution of a Thermophilic Anaerobe Dissulfurirhabdus thermomarina SH388T Involved in Disproportionation of Sulfur Compounds in Shallow Sea Hydrothermal Vents
Source: Microorganisms. 2020 Jul 27;8(8):1132. doi: 10.3390/microorganisms8081132 (PMC7463578; doi:10.3390/microorganisms8081132)
Supplement: Supplementary file 1 [file microorganisms-08-01132-s001.pdf]

microorganisms

Supplementary material

Genomic characterization and environmental distribution of a thermophilic anaerobe *Dissulfurirhabdus thermomarina* SH388^T involved in disproportionation of sulfur compounds in shallow-sea hydrothermal vents

Maxime Allioux [1], Stéven Yvenou [1], Galina Slobodkina [2], Alexander Slobodkin [2], Zongze Shao [3], Mohamed Jebbar [1] and Karine Alain [1,*]

[1] Univ Brest, CNRS, IFREMER, LIA1211, Laboratoire de Microbiologie des Environnements Extrêmes LM2E, IUEM, Rue Dumont d'Urville, F-29280 Plouzané, France; Maxime.Allioux@univ-brest.fr (M.A.); steven.yvenou@gmail.com (S.Y.); Mohamed.Jebbar@univ-brest.fr (M.J.); Karine.Alain@univ-brest.fr (K.A.)
[2] Winogradsky Institute of Microbiology, Research Center of Biotechnology of the Russian Academy of Sciences, 117312 Moscow, Russia; gslobodkina@mail.ru (G.S.); aslobodkin@hotmail.com (A.S.)
[3] Key Laboratory of Marine Genetic Resources, Third Institute of Oceanography, Ministry of Natural Resources, Xiamen 361005, China; shaozongze@tio.org.cn (Z.S.)
* Correspondence: Karine.Alain@univ-brest.fr

Supplementary Material S1: *Dissulfurirhabdus thermomarina* SH388^T genome locus tags, genomic islands composition, and results of comparative genomics.

S1.1. Synthesis of the gene loci

Table S1.1. Correspondences between the loci of the annotations by Prokka, Dfast, RAST, PGAP (2020-03-30.build4489) and UniProtKB with the CDSs of the NCBI's online automated prokaryotic genome annotation pipeline. CDSs found with their associated loci, based on the assembly repository ASM1297923v1 (Abbreviation: NR, not retrieved).

Gene name	Gene associated Locus (NCBI PGAP)
**Gene related to carbon metabolism:**	
**Wood-Ljundhal**	
Formate dehydrogenase	HCU62_RS06340
formate--tetrahydrofolate ligase	HCU62_RS06190
FolD bifunctional 5,10-methylene-tetrahydrofolate dehydrogenase/cyclohydrolase	HCU62_RS06185
5-methyltetrahydrofolate:corrinoid/iron-sulfur protein co-methyltransferase	HCU62_RS06135
5,10-methylenetetrahydrofolate reductase	HCU62_RS06150
carbon monoxide dehydrogenase	HCU62_RS06180
Acetyl-CoA decarbonylase/synthase	HCU62_RS06140 ; HCU62_RS06145
**Glycolysis**	
glucose-6-phosphate isomerase	HCU62_RS09635
fructose-1,6-bisphosphatase	HCU62_RS01815
ATP-dependent 6-phosphofructokinase	HCU62_RS01170
fructose-bisphosphate aldolase	HCU62_RS04890
triose-phosphate isomerase	HCU62_RS06960
glyceraldehyde-3-phosphate dehydrogenase	HCU62_RS06940
phosphoglycerate kinase	HCU62_RS06955
2,3-bisphosphoglycerate-independent phosphoglycerate mutase	HCU62_RS01330 ; HCU62_RS03440
phosphopyruvate hydratase	HCU62_RS01970
phosphoenolpyruvate synthase	HCU62_RS09245
pyruvate kinase	HCU62_RS10405
**Glycogen degradation**	
glycogen phosphorylase	HCU62_RS04055
alpha-D-glucose phosphate-specific phosphoglucomutase	HCU62_RS05730
**Pyruvate fermentation to acetate**	
acetate—CoA ligase	HCU62_RS09440
pyruvate synthase	HCU62_RS00790 ; HCU62_RS00785 ; HCU62_RS00800
**Pentose phosphate pathway**	
glucose-6-phosphate dehydrogenase	HCU62_RS08000
6-phosphogluconolactonase	HCU62_RS07995
6-phosphogluconate dehydrogenase	HCU62_RS08005
ribose-5-phosphate isomerase	HCU62_RS01010
ribulose-phosphate 3-epimerase	HCU62_RS08705
transketolase (glycolaldehydetransferase)	HCU62_RS03690
transaldolase (dihydroxyacetonetransferase)	HCU62_RS08675
**Formate associated enzymes**	
formate dehydrogenase	HCU62_RS01485 ; HCU62_RS06125 ; HCU62_RS06340
formate—tetrahydrofolate ligase	HCU62_RS06190

Gene name	Gene associated Locus (NCBI PGAP)
**Genes related to hydrogen metabolism:**	
methyl-viologen-reducing hydrogenase delta subunit/coenzyme F420-reducing hydrogenase delta subunit	HCU62_RS00680 ; HCU62_RS03360 ; HCU62_RS06160 ; HCU62_RS06170 ; HCU62_RS06335
HypC/HybG/HupF family hydrogenase formation chaperone	HCU62_RS01665
hydrogenase formation protein HypD	HCU62_RS01670
hydrogenase expression/formation protein HypE	HCU62_RS01675
hydrogenase maturation protease	HCU62_RS01680
hydrogenase maturation nickel metallochaperone HypA	HCU62_RS01685
hydrogenase nickel incorporation protein HypB	HCU62_RS01690
hydrogenase-2 small chain	HCU62_RS02130
nickel-dependent hydrogenase large subunit	HCU62_RS02135
Ni/Fe-hydrogenase, b-type cytochrome subunit	HCU62_RS02140
**Genes related to nitrogen metabolism:**	
hydroxylamine oxidase	HCU62_RS03110
hydroxylamine reductase	HCU62_RS07985
ammonia transporter	HCU62_02830
P-II family nitrogen regulator	HCU62_RS02830 ; HCU62_RS02845
**Genes related to sulfur metabolism:**	
sulfate adenylyltransferase	HCU62_RS05615 ; HCU62_RS05700
adenylyl-sulfate reductase subunit A	HCU62_RS05605
adenylyl-sulfate reductase subunit B	HCU62_RS05610
manganese-dependent inorganic pyrophosphatase	HCU62_RS08685
dissimilatory sulfite reductase subunits alpha	HCU62_RS08555
dissimilatory sulfite reductase subunits beta	HCU62_RS08560
dissimilatory sulfite reductase subunits gamma	HCU62_RS09615
dissimilatory sulfite reductase D	HCU62_RS08565
DsrM	HCU62_RS08595
DsrK	HCU62_RS08600
DsrJ	HCU62_RS08605
DsrO	HCU62_RS08610
DsrP	HCU62_RS08615
QmoA	HCU62_RS05600
QmoB	HCU62_RS05595
QmoC	HCU62_RS05590
sulfurtransferase TusA family protein	HCU62_RS03365 ; HCU62_RS06620
sulfurtransferase	HCU62_RS06555 ; HCU62_RS06560
polysulfide reductase NrfD	HCU62_RS00235 ; HCU62_RS11490 ; HCU62_RS11500
polysulfide/tetrathionate/thiosulfate reductase associated proteins	HCU62_RS07540 ; NR
TorD-like chaperon protein	HCU62_RS07530
molybdopterin oxidoreductase	HCU62_RS06125 ; HCU62_RS07535 ; HCU62_RS07580 ; HCU62_RS10115
rhodanese-like domain-containing protein	HCU62_RS02325 ; HCU62_07590
putative thiosulfate sulfurtransferase	HCU62_RS06560

S1.2. Annotation of Genes of Genomic Islands

Table S1.2. Gene annotations within the genomic islands (GI) of *Dissulfurirhabdus thermomarina* SH388^T, based on the IslandViewer 4 webserver.

Genomic island	Gene start	Gene end	Strand	Product
GI 1	1793219	1795750	1	sugar ABC transporter substrate-binding protein
GI 1	1795761	1796717	1	LPS biosynthesis protein
GI 1	1796736	1797908	1	hypothetical protein
GI 1	1797905	1799242	1	polysaccharide biosynthesis related protein
GI 1	1799525	1800247	1	hypothetical protein
GI 1	1800508	1801404	1	sulfotransferase family protein
GI 1	1802691	1803914	1	glycosyl transferase family 1
GI 1	1803904	1804683	1	acetyl-mannosamine transferase
GI 1	1804791	1805603	1	hypothetical protein
GI 1	1805600	1806730	1	GDP-mannose 4,6-dehydratase
GI 1	1806768	1807709	1	GDP-L-fucose synthase
GI 1	1807723	1809150	1	mannose-6-phosphate isomerase
GI 1	1809117	1810670	-1	Phosphomannomutase
GI 2	1867701	1869266	1	hypothetical protein
GI 2	1870044	1870256	-1	hypothetical protein
GI 2	1870376	1870660	1	hypothetical protein
GI 2	1870586	1871059	-1	hypothetical protein
GI 2	1871214	1872170	1	IS30 family transposase
GI 2	1872355	1872564	-1	hypothetical protein
GI 2	1872561	1873196	-1	hypothetical protein
GI 2	1875044	1875547	-1	hypothetical protein
GI 2	1875917	1876378	-1	hypothetical protein
GI 2	1876378	1876983	-1	hypothetical protein
GI 2	1877128	1877793	-1	hypothetical protein
GI 2	1878138	1878791	-1	hypothetical protein
GI 2	1878926	1879153	-1	hypothetical protein
GI 2	1881275	1882240	1	hypothetical protein
GI 2	1882711	1884291	-1	RNA polymerase sigma factor RpoD

S1.3. Comparative genomics

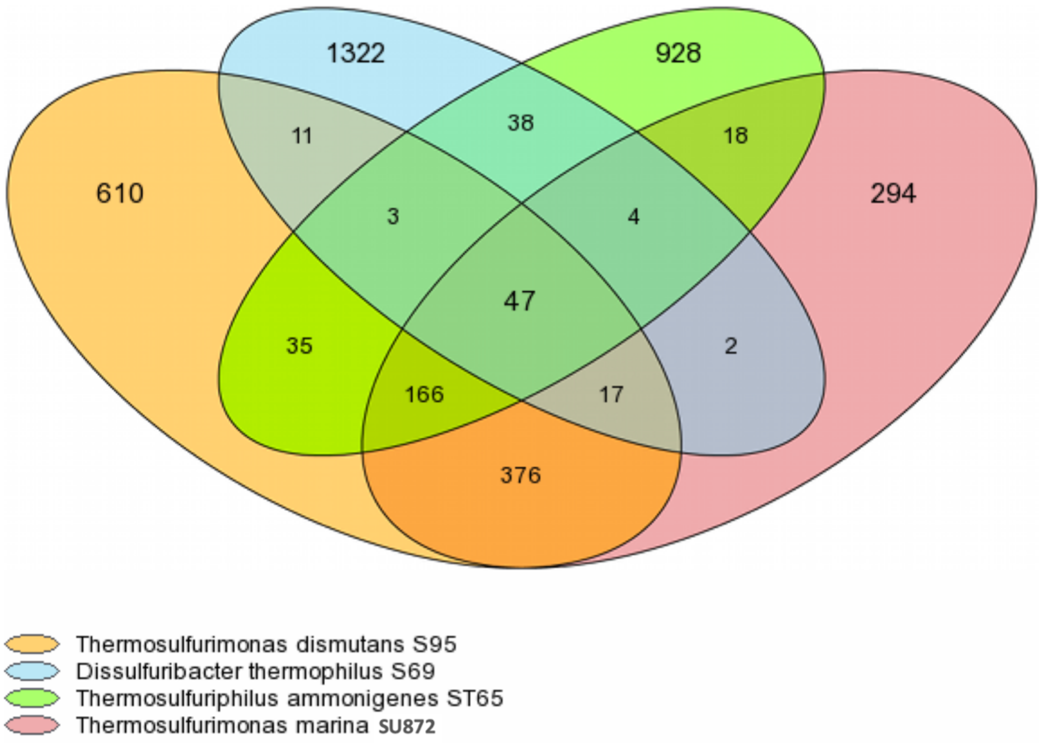

Figure S1.3. Homologous CDSs found by comparative genomics with the MaGE platform among the genomes of the hydrothermal bacteria *Thermosulfurimonas marina*, *Thermosulfuriphilus ammonigenes*, *Dissulfuribacter thermophilus*, and *Thermosulfurimonas dismutans*, excluding homologous CDSs from the genome of *D. thermomarina*.
